# Coumarin–Amino Acid Hybrids Used as Possible Multifactorial Anti-Inflammatory Agents

**DOI:** 10.3390/ijms27104443

**Published:** 2026-05-15

**Authors:** Ioannis Fotopoulos, Dimitra Hadjipavlou-Litina

**Affiliations:** Laboratory of Pharmaceutical Chemistry, School of Pharmacy, Faculty of Health Sciences, Aristotle University of Thessaloniki, 54124 Thessaloniki, Greece

**Keywords:** LOX, COX-2, inflammation, coumarins, amino acids, hybridization

## Abstract

A series of coumarin–amino acid hybrids of glycine, γ-aminobutyric acid, and *L*-glutamic acid was developed. These compounds were evaluated for their antioxidant and anti-inflammatory activities *in vitro* and for their drug-likeness *in silico*. Antioxidant activity was assessed *in vitro* using the AAPH-induced linoleic acid peroxidation assay. Soybean lipoxygenase and ovine cyclooxygenase 2 were used *in vitro* to test the inhibitory activity of the adducts. An *in silico* evaluation was performed using the open-access platforms Molinspiration, SwissADME, PreADMET, Molsoft, GLORYx, CypRules, and LiverTox Workspace. The synthesis of the compounds proceeded via a facile procedure through the corresponding acid in very good yields. The antioxidant activity of the compounds is shown to be highly dependent on the linkage used, with compound **15** presenting the highest activity (93% inhibition). The most active LOX inhibitor is compound **4** (IC_50_ = 58 μM), while compounds **4** and **5** are the most potent COX-2 inhibitors (IC_50_ = 55 μM for both). Compounds **4, 9**, and **15** are depicted as pleiotropic molecules (compound **4**: IC_50_ for SLOX-1 = 58 μM and IC_50_ for COX-2 = 55 μM; compound **9**: IC_50_ for COX-2 = 60 μM, and 59% antilipid peroxidation; compound **15**: IC_50_ for COX-2 = 70.5 μM, 93% antilipid peroxidation). An *in silico* evaluation showed favorable properties of the designed agents, which were quantified, with all the compounds showing a QED score higher than 0.5. The overall results highlight that compound **4** can be used as a lead molecule for the design of more potent agents with a pleiotropic profile.

## 1. Introduction

Inflammation is the response of an organism to various stimuli. It triggers the immune system to respond and restore the tissue’s functionality. *Lipoxygenases* and *Cyclooxygenases* are enzymes implicated in the arachidonic acid cascade. Arachidonic acid (5Z,8Z,11Z,14Z)-icosa-5,8,11,14-tetraenoic acid) is a polyunsaturated fatty acid (PUFA) that is tagged on the inner cellular membrane by its phospholipid form. Upon stimulation, phospholipase A_2_ (PLA_2_) is activated and releases free acid from the cellular membrane [1]. The release of arachidonic acid triggers lipoxygenase and cyclooxygenase enzymatic activity, leading to a series of inflammatory mediators.

In mammals, six different LOX isoforms have been identified: 5-LOX, 12-LOX, 12/15LOX, 15-LOX type 2, 12*R*-LOX, and epidermal LOX [2]. They can incorporate a molecule of oxygen in the substrate to yield hydroperoxides, keto-lipids, and epoxy leukotrienes as final products. In humans, the major COX isoforms are COX-1 and COX-2. These heme-containing homodimeric proteins possess dual catalytic activity by inserting, firstly, two molecules of oxygen into arachidonic acid to yield prostaglandin G_2_ (PGG_2_), and secondly, by inducing a peroxidation reaction on PGG_2_ to produce prostaglandin H_2_ (PGH_2_). COX-1 is consistently expressed in cells and presents a protective role in controlling the physiological processes of the organism. On the contrary, COX-2 is an isoform that is expressed at low levels, and, upon stimulation of the cell, its expression is significantly upregulated, indicating a high correlation to inflammation.

PGH_2_ has a short half-life [3], acting as a substrate for a plethora of other enzymes, and it can be transformed into many different transmitters with pro- and anti-inflammatory properties. Thromboxane A_2_ (TXA_2_) is one of the most important products of PGH_2_ metabolism, derived from TXA_2_ synthase activity [4]. As a result, it is evident that the inhibition of COX-2 will offer significant anti-inflammatory activity.

The brain is an organ important for the physiological functionality of the human body. PUFA metabolism is of paramount importance to the brain. The main amino acid neurotransmitters are glycine, gamma-aminobutyric acid (GABA), and glutamic acid. GABA exerts a sedative activity in the adult brain, while glutamic acid plays an excitatory role. On the other hand, glycine, depending on the receptor, exerts both activities [5,6]. The relation between sedative and excitatory neurotransmission is tightly controlled because the smallest disruption can lead to high Ca^2+^ influx to the cell [7], which activates the pro-inflammatory enzymes 5-LOX and COX-2.

It has been documented that PGE_2_ lowers the ability of glycine to bind to its receptors [8] and that the prolonged release of glutamic acid in the synaptic cleft leads to an increase in the intracellular Ca^2+^ concentration, inducing a further increase in glutamic acid in the cleft [9,10]. This phenomenon is termed “excitotoxicity” and is tightly related to the nerve cells’ functionality. The above-mentioned data highly support the direct correlation between the control of neurotransmission and inflammation in the brain. COX-2 is also tightly linked to excitotoxicity. It has been documented that COX-2 induces the release of glutamic acid in the synaptic cleft [11], disrupting the proper function of the blood–brain barrier (BBB) [12], and that COX-2 expression is enhanced in neurons under excitotoxic conditions [13]. Therefore, inhibition of COX-2 is considered of significant interest.

Over the last decade, a new type of cell death—common in the CNS—has been documented. This type of cell death is directly linked to iron levels and is termed “ferroptosis” [14]. PUFAs are very important for brain functionality because they regulate CNS plasticity [15] and facilitate neurotransmitter release [16]. Fe^2+^ and Fe^3+^ ions induce pro-inflammatory free radical species as well as reactive oxygen and nitrogen species (ROS and RNS, respectively). 5-LOX [17] and COX-2 [18] were found to facilitate ferroptosis. LOX, via its products of lipid peroxidation, is implicated with the execution of ferroptosis [19,20]. Although non-enzymatic autooxidation of PUFAs seems to be the main source of ferroptosis progression, the activity of LOX as an inflammation-induced enzyme increases the levels of peroxy radicals, which contribute significantly to ferroptosis. COX-2 is linked to ferroptosis by the induction of PTGS2, which encodes COX-2. As a result, the end-products of arachidonic acid metabolism by COX-2 increase and lead to inflammation [21]. Considering these, inhibition of 5-LOX and COX-2 is significant when treating neuro-inflammatory diseases.

Nature is, undoubtedly, the biggest source of inspiration for a medicinal chemist to design and synthesize bioactive molecules with excellent enzyme specificity and low toxicity. Coumarins (or 2*H*-benzopyrones) are a class of natural products abundant in the *Rutaceae, Umbelliferae*, and *Orchidaceae* families. Several reports highlight the antimicrobial [22], antioxidant [23] and anti-inflammatory [24] potential of coumarin derivatives. Their anti-inflammatory activities can be attributed to their COX and LOX inhibition, to their ability to induce phagocytosis, and to the inhibition of inducible Nitric Oxide Synthase (iNOS) [25,26,27]. Examples of natural coumarin LOX inhibitors are esculetin, fraxetin, and daphnetin [27]. Several reports describe the promising activity of coumarin derivatives on COX-2 [28]. They possess negligible toxicity in humans; thus, they can be safely used as a privileged scaffold for the design of bioactive compounds [29].

Coumarin hybrids have received recent attention from the scientific community regarding their anti-cancer potential, especially their activity against breast cancer, acting, as, e.g., caspase-9 activators to induce apoptosis [30,31], inhibitors of VEGFR-2 [32] to inhibit angiogenesis, or even as p38a and MAPK inhibitors. The reader can refer to the review papers of Yadav et al. [33] and Hacholli et al. [34] to gain more in-depth knowledge regarding the mechanism of anti-cancer activity of coumarin hybrids.

Molecular hybridization is a synthetic strategy that combines moieties or pharmacophores from different bioactive molecules into a single chemical entity, furnishing a hybrid molecule that acts as a multitarget drug on multifactorial diseases, with improved efficacy, better selectivity, less toxicity, and reduced side effects compared to the parent drugs [35,36,37]. Neurodegenerative diseases, such as Alzheimer’s or Parkinson’s disease, are considered multifactorial. Oxidative stress (i.e., the production and accumulation of reactive oxygen species) is thought to be highly related to the onset and the induction of these diseases [38].

**Figure 1:**
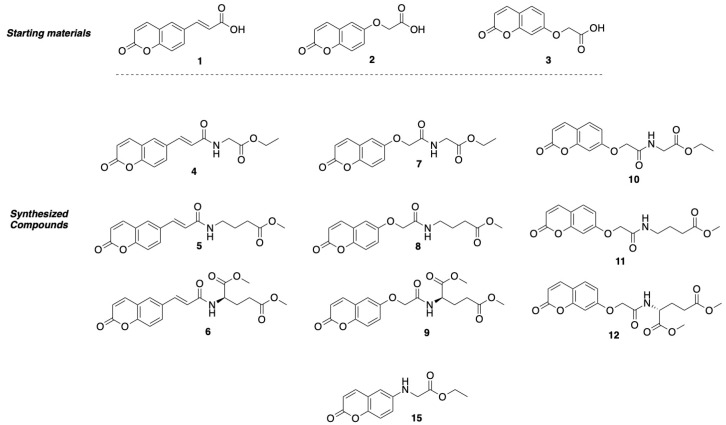
Chemical structures of starting materials and synthesized hybrids studied in this work.

The design of dual LOX and COX-2 inhibitors can be considered as one step closer to developing anti-inflammatory agents. If this activity is further combined with the documented antioxidant and neuroprotective action of glycine [39,40], as well as with its neuroprotective activity [41], along with GABA’s inhibitory role in autoimmune inflammation within the CNS [42,43] and the antioxidant activity of glutamic acid [44,45], one can successfully develop molecules against neuro-inflammatory conditions. Continuing on with our previous work on anti-inflammatory agents [46], we present herein the design and synthesis of a series of hybrids bearing the coumarin moiety and the neuromodulator amino acids glycine, GABA, and glutamic acid, as well as their *in silico* drug-likeness and their ADMET properties, and their *in vitro* evaluation as inhibitors of lipid peroxidation and LOX and COX-2 enzymes. The structures of the designed compounds are given in Figure 1.

## 2. Results and Discussion

### 2.1. Chemistry

For the synthesis of compound **1**, a Knoevenagel–Doebner reaction between 2H-chromen-6-carboxaldehyde and malonic acid was implemented. The final compound was isolated in high yield (76%). Compounds **2** [2-((2-oxo-2H-chromen-6-yl)oxy)acetic acid] and **3** [2-((2-oxo-2H-chromen-7-yl)oxy)acetic acid] were synthesized previously by our group [47] and were used without any further purification. The acrylic amides (4, 5, and 6) were synthesized in good yields (58–100%) while the oxo derivatives (7–12) were in medium yields (39–75%). Despite the medium yields, this synthetic procedure is considered the most common in the literature due to its facile workup and its low probability for epimerization [48,49]. The synthesis of compound **15** [ethyl (2-oxo-2H-chromen-6-yl)glycinate] showed low yield (a 27% overall yield for the three-step procedure), mainly due to the difficulty of crystallization of the desired compound.

The structure of the synthesized compounds was verified using ^1^H-NMR, ^13^C-NMR, and HRMS techniques. Regarding the acrylic derivatives, ^1^H–NMR showed the characteristic coupling constant for vinylic protons (*J* = 15.8 Hz). The solvent used for the NMR experiment affected the amide proton peak type, which, in CDCl_3_, appeared as a singlet, while in DMSO-d_6_, it appeared as a triplet, significantly downfield. The HRMS data fully agree with the proposed structure that there is a difference between the theoretical and experimental values of the specific ion of less than 5 ppm.

### 2.2. Biology

In this research, acids and new hybrids were evaluated as multitarget agents, i.e., inhibitors of soybean lipoxygenase, ovine COX-2, and lipid peroxidation (which resembles the lipid peroxidation of membranes). The compounds are stable under the experimental conditions used. The results of the biological evaluation of the hybrids are given in Table 1.

#### 2.2.1. AAPH-Induced Peroxidation Inhibition Assay

ROS are highly reactive, and some are characterized as extremely toxic. This reactivity/toxicity leads to pathological conditions. Neuroinflammation is characterized by a significant enhancement of lipid peroxidation, localized in the Alzheimer’s patient’s brain. Thus, antioxidants with brain antilipid peroxidation activity could offer therapy. We decided to determine the hybrids’ antioxidant activity in comparison to Trolox, a well-known antioxidant reference compound. 2,2-azobis-(2-amidinopropane) hydrochloride (AAPH) was used to generate *in vitro* peroxy radicals through thermal decomposition at 37 °C under atmospheric oxygen. The results are described based on the structural characteristics of the hybrids.

The main differences among the structural characteristics are related to the nature of the acid: (i) acrylic acid and (ii) oxo acid. For the latter, the oxo position is either 6-oxo or 7-oxo. Another point of structural change is the selected amino acid. Among the acids 1, 2, and 3, only the 6-oxo acid 2 exhibited antilipid peroxidation activity, whereas for the amides of the 6-coumarin-acrylic acid with glycine and L-glutamate, they presented moderate results (37% and 23%, respectively). The GABA led to an inactive derivative.

We tried to compare the responses among the amides of the acrylic acid and of the 6-oxo acid. The glycinate derivative 4 is almost equipotent to the corresponding 7 (37%/39%). On the contrary, GABA derivative 5 does not present any activity compared to derivative 8 by 32%. Furthermore, the glutamate derivative 6 is less potent (23%) than 9 (59%).

Considering the 6-oxo-derivatives, the antioxidant activity ranged from 32 to 59%, with glutamate amide being more potent. The results of the 7-oxo amides showed low activity (13–28%, compound 10–12). Judging the three subgroups, the results were more promising for the 6-oxo substituted amides, and especially for the glycinates and glutamates.

#### 2.2.2. Soybean Lipoxygenase Inhibitory Activity Assay

The role of 5-LOX as an endogenous modulator of neurological disorders has been well defined [50]. 5-LOX protein levels have been found to be higher in AD brains, compared to healthy controls. Thus, the inhibition of 5-LOX will be beneficial for neuroinflammation and AD. In our tests, we used SLOX-1 isoenzyme, which uses linoleic acid as a substrate, with maximum activity in pH = 9 [51]. SOX-1 produces a conjugated diene absorbing at 234 nm, converting linoleic acid into 13-hydroperoxylinoleic acid. Experimental results showed a sufficient qualitative correlation between the inhibition values of the purified human LOX enzyme and SLOX-1 [52]. While one may not extrapolate the quantitative results of this assay to the inhibition of mammalian 5-LOX, it has been shown that inhibition of plant LOX activity by NSAIDs is qualitatively quite similar to the inhibition observed on rat mast cells LOX, and this assay may be used as a qualitative or semi-quantitative screening for such activity. Both share similar structural domains (a, β-barrel N-terminal domain and a C-terminal catalytic domain). Reddy et al. [53] compared binding sites of soybean lipoxygenase with human 5- LOX and reported that both share similarity with each other. The sequence identity between plant and mammalian lipoxygenases is highest in the portions of the catalytic domain near the iron atom [54]. Manjunath et al. [55] also compared amino acid binding sites in human 5-LOX and soybean 3-LOX by comparing the physicochemical properties using the MultiBind program, supporting their similarities. Nordihydrogouaretic acid (NDGA) was used as a reference compound. The results of the biological evaluation of the hybrids are given in Table 1.

Among the acids 1, 2, and 3, it seems that the oxo-coumarinic acids 2 and 3 are equipotent, presenting an IC_50_ value of 100 μM, and are more potent than acid 1. For acrylic acid 1, very low inhibition was noticed by the glutamate and gaba amides 5 and 6, whereas glycinate analog 4 was found significantly potent, with an IC_50_ value of 58 μM. Furthermore, comparing the derivatives of 6-acrylic acid to the 6-oxo, it seems that 4 is significantly active, whereas 7 is inactive under the reported conditions. Compound **5** presents 41% inhibition, whereas 8 does not have any activity. Hybrid 6 exhibits medium inhibition, whereas 9 has an IC_50_ value of 100 µM. Among the 6-oxo amides, only glutamate 9 exhibited a significant inhibitory activity (IC_50_ = 100 μΜ), whereas no activity was recorded for the glycinate (compound **7**) and the gaba (compound **8**) amides. No activity was also recorded for the 7-oxo amide derivatives 10–12; compound **15** exerted low activity (29% inhibition).

#### 2.2.3. Ovine Cyclooxygenase 2 (COX-2) Inhibitory Activity Assay

The pathogenesis of neurological disorders is widely associated with COX-2. As a result, hybrid derivatives were evaluated for their COX-2 inhibitory activity *in vitro*. The results of the biological evaluation of the hybrids are given in Table 1.

In our research, we used ovine COX-2. *In vitro* protocols can be classified into two distinct groups: assays using enzymes or cells. Enzymatic methods use purified or recombinant enzymes [56] while cellular models use various mammalian cell types [57,58]. Ovine and human COX-2 enzymes show about 90% sequence homology [59], and minor differences in the sequences between species can modify the drug–enzyme interaction. They are both homodimeric enzymes (~72 kDa per subunit) and function as monotopic membrane proteins anchored to the endoplasmic reticulum. Human COX-2 features a specific Ile523Val substitution (Val in human, Ile in ovine-1) that increases the volume of the binding site, allowing for the binding of more bulky selective inhibitors (coxibs).

No inhibition was found for the acids 1, 2, and 3. However, amides 4 and 5 exhibited equipotent COX-2 inhibition. Although both the acids and the amino acids used for hybridization are different, the final inhibition results were found to be similar, possibly due to the position taken by these molecules in the enzyme active site.

Among the 6-oxo derivatives 7–9, the glutamate derivative 9 gave an IC_50_ value of 100 μM. No activity was recorded for the glycinate derivative 7, while the gaba amide 8 exerted 44% inhibition. For the 7-oxo derivatives 10–12, very low to no activity was recorded. Interesting results were obtained from amide derivative 15, which exerted an IC_50_ value of 70.5 μM.

Considering our results, compounds **4**, **5**, and **9** can be considered as dual COX-2/LOX inhibitors. Although their activity is significantly lower than that of the reference compounds, NDGA and indomethacin for LOX and COX-2, respectively, their inhibitory values and pharmacokinetic profiles can be used to modify their structure to develop significantly more potent agents. This would add to their therapeutic importance, as compounds with dual activity against two well-established inflammatory targets will exert a better profile (e.g., fewer drug–drug interactions, better pharmacokinetic profile) than the use of two different inhibitors of LOX and COX-2. Furthermore, compound **15**, with high antilipid peroxidation behavior and COX-2 inhibition, can also be considered as a multifunctional agent. Furthermore, comparing the derivatives of 6-acrylic acid to the 6-oxo, it seems that 4 is significantly active, whereas 7 is inactive under the reported conditions. Compound **5** presents an IC_50_ of 55 µM, whereas 8 had 44% inhibition. Hybrid 6 exhibits no inhibition, whereas 9 has an IC_50_ value of 60 µM. It should be noted that compounds **4**, **5**, and **9** did not present any COX-1 inhibition in the preliminary *in vitro* assays.

### 2.3. Computational Experiments

The pharmacokinetic and physicochemical properties of the designed compounds were evaluated *in silico.* The compounds (Figure S1, supplementary section) were qualitatively studied for their “drug-likeness”. Their blood–brain barrier (BBB) permeability, P-glycoprotein (Pgp) substrate potentiality, metabolic pathways, hepatotoxicity, and Cytochrome P450 (CYP) interactions were predicted accordingly. Furthermore, the overall drug-likeness was quantified by calculating their corresponding QED score [60]. The results are given in the supplementary data section.

A physicochemical study (Table S1, supplementary section) of the compounds showed that none violated the Ro5 criteria. Regarding BBB penetration (Table S2, supplementary section), the designed molecules cannot penetrate the BBB via passive diffusion (SwissADME data), except for the amine derivative 15. The hybrid amino acid–coumarin structure may facilitate penetration through active transport using the amino acids’ transporters. To our knowledge, there are no computational tools in the literature that could be used to evaluate this alternative. BBB predicted penetration data, received by PreADMET, showed low values. The data retrieved from the MolSoft platform indicates that all the compounds partly penetrate the BBB via diffusion since their BBB score values are around 3. Given the higher robustness of the SwissADME platform, it is considered the most reliable tool for lead optimization in the design of better BBB-penetrating agents in predicting ADME properties of compounds [61].

The Pgp interaction data (Table S2, supplementary section, SwissADME and PreADMET columns) show that none of the studied molecules is a substrate for the Pgp protein, indicating that once these agents are delivered to the brain, they are most likely to remain within. The only exception to the overlap of the two tools is compound 9. In the PreADMET column, it seems that compound 9 is a substrate for the Pgp protein. From the gastrointestinal absorption data (Table S3, supplementary section), it is highlighted that all the studied compounds are readily absorbed and show a protein binding percentage above 50%. Warfarin was selected as a reference compound, which is known to be readily absorbed following per os administration.

Regarding the predicted *in silico* results of their metabolism (Table S4, supplementary section), it is evident that the acrylate derivatives 4–6 follow a Phase II metabolism, being conjugated with glutathione (medium probability 0.37–0.48). Other routes include the hydrolysis of the ester moiety of the amino acid. The replacement of the acrylic core with the oxoacetate moiety (compounds 7–12) resulted solely in a Phase I metabolism either by hydrolysis of the ester moiety or by hydroxylation of the coumarin core at position 3. This is in accordance with the bibliographic data [62]. The same pattern is followed by the amine derivative 15. Prediction is validated by paracetamol and midazolam used as reference compounds. Paracetamol in humans follows solely Phase II, while midazolam is solely metabolized via Phase I.

A drug acting as an inhibitor or an inducer of a CYP isoform can alter the efficacy of other co-administered drugs. The CypRules platform was used to predict the behavior of the developed hybrids. The results (Table S5, supplementary section) showed that none of the evaluated compounds act as a cytochrome P-450 inhibitor. The most important isoforms were selected, namely CYP1A2, CYP2C19, CYP2C9, CYP2D6, and CYP3A4. As reference compounds, cimetidine (a known CYP1A2, CYP2C19, and CYP3A4 inhibitor) and fluoxetine (a known CYP2C9 and CYP3A4 inhibitor) were used.

The predicted hepatotoxicity data (Tables S6 and S7, supplementary section) show the capability of the compounds to induce liver damage. Most of them were predicted as responsible for hyperbilirubinemia and cholestasis. All the compounds studied showed at least three interactions with the evaluated transporters. Pgp transport data are in accordance with the data in Table S2 (supplementary section). Ximelagatran was used as a reference compound due to its discontinued status [63] based on its documented hepatotoxicity. Ximelagatran exerted interactions with ten of the selected transporters.

In an attempt to quantify the drug-likeness of the designed compounds, the QED score was used [60]. The results can be viewed in three different ways: overall, all the compounds exert promising drug-likeness, as concluded by their respective QED scores, which are above 0.5 and are very close (or even exceed) the QED scores of the reference compounds NDGA and indomethacin. Compound **15** showed a QED score that is only 3% lower than that of indomethacin. Examining the results based on the amino acid moiety, the glycinates are more drug-like molecules than their counterparts, with compound **15** having the highest score of 0.866. This can be partially attributed to the compound’s small size, which enables further structural modification to increase its drug-likeness. Regarding the linker used between the coumarin and the amino acid core, the results do not show any statistically important decrease in the QED scores.

### 2.4. Prediction of Anti-Inflammatory Activity

Our extended research and experience in QSAR studies on non-steroidal anti-inflammatories [64] and on the in vivo investigation of the anti-inflammatory activity of cinnamic acid derivatives and hybrids helped us to predict, using the appropriate validated models, the in vivo anti-inflammatory activity of the most interesting compound **4**. We considered the model of carrageenin-induced rat paw edema (CPE) in rats a prestigious assay for determining anti-inflammatory activity.

The following QSAR model [64] was used to predict in vivo activity. Using the clogP value of 1.33 [65], the anti-inflammatory activity was predicted to be 44%.

log CPE% = 0.053((0.036) c log P + 1.543((0.122))

n = 6; r = 0.898; r^2^ = 0.806; q^2^ = 0.584; s = 0.046; F_1,4_ = 15.56; α = 0.05

The regression equation incorporates 95% confidence intervals, the correlation coefficient (r), the standard deviation (s), the cross-validated correlation coefficient squared (q^2^), and F-values for each individual term. The predicted ADMET properties of compound **4**: Absorption = High, % protein binding = 82.49%, TPSA = 85.61, Bioavailability = 0.55, leadlikeness = Yes (by Lipinski, Veber, Ghose, Egan, Muegge rules), as well as the QED score of 0.836, support the adequate absorption per os and bioavailability (Table S8, Supplementary Material). Compound **4** presents excellent membrane permeability, with Caco-2 and MDCK values well above high-permeability. The predicted human oral absorption suggests permeability-driven uptake.

## 3. Materials and Methods

### 3.1. Chemistry

#### 3.1.1. Instrumentation

The melting points were recorded on a Mel-Temp II (Laboratory Devices, Holliston, MA, USA) device and are given uncorrected. The infrared (IR) spectra were recorded on a single-beam Perkin-Elmer Spectrum BX FT-IR (710 Bridgeport Ave, Shelton, CT, USA) apparatus as KBr disks. Nuclear Magnetic Resonance (NMR) spectra were recorded on a Bruker Avance 400 (40 Manning Road, Billerica, MA, USA) (400 MHz for ^1^H and 100 MHz for ^13^C nuclei, respectively) or on an Agilent 500/54 DD2 (5301 Stevens Creek Blvd, Santa Clara, CA, USA) (500 MHz for ^1^H and 125 MHz for ^13^C nuclei, respectively) apparatus, and it is stated accordingly. Tetramethylsilane (TMS) was used as an internal standard, and its chemical shift is given as zero (δ_TMS_ = 0). The solvent used each time is stated in each experiment. The following abbreviations for the peaks are used: s = singlet, brs = broad singlet, d = doublet, dd = doublet of doublets, t = triplet, q = quartet, quint = quintet, td = triplet of doublets, and m = multiplet. High-resolution mass spectra (HRMS) were recorded on an Agilent Q-TOF G6540B with a dual AJS ESI-MS apparatus. Liquid chromatography spectra (LC–MS) were recorded on a Shimadzu LCMS-2010 EV spectrometer (1, Nishinokyo Kuwabara-cho, Kyoto, Japan). Optical activity was recorded on an A. Kruss Optonic apparatus (Zollhallenstraße 11, Freiburg, Germany) in a 50 mm cuvette. Thin-layer chromatography (TLC) was performed on F_254_ plaques (Merck), and ultraviolet (UV) light was used as a visualizing agent. Column chromatography was performed on 230–400 mesh silica gel (Merck).

#### 3.1.2. Chemical Reagents

All chemicals were supplied by Merck (Darmstadt, Germany), Fluorochem (Derbyshire, UK), and Alfa Aesar (Kandel, Germany) and used as supplied. 2-oxo-2*H*-chromen-6-carboxaldehyde was purchased from Flurochem (Derbyshire, UK). Coumarin-6 (and 7)-yloxyacetic acids were previously synthesized by our group and were used in this work without further purification. The solvents used were of reagent grade, and anhydrous solvents were obtained by performing distillation upon proper drying agents.

#### 3.1.3. Synthesis of (E)–(3–(2–Oxo-2H-Chromen-6-yl)) Acrylic Acid (1) [66]

**Figure 2:**
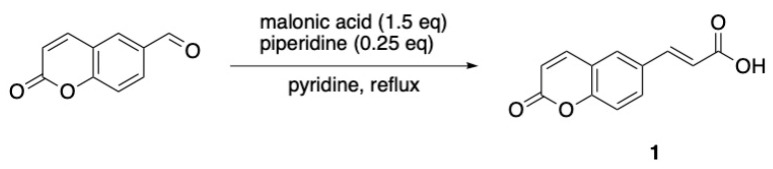
Synthesis of compound **1**.

In a round-bottomed flask containing 10 mL of pyridine, 500 mg (2.87 mmol, 1 eq) of 2-oxo-2*H*-chromen-6-carboxaldehyde was dissolved at 0 °C, and the mixture was stirred using a magnetic stirrer. Subsequently, 449 mg (4.31 mmol, 1.5 eq) of malonic acid and 70.9 μL (0.718 mmol, 0.25 eq) of piperidine were added, and the mixture was refluxed. Upon completion of the reaction (TLC monitoring), 2M HCl solution was added until pH = 1. The formed solid was filtered off and recrystallized from water, yielding a beige—colored solid in good yield (Figure 2).

##### (*E*)–(3–(2–Oxo-2H-Chromen-6-yl))Acrylic Acid (1)

Beige-colored solid. Yield: 76%. R_f_ (2PS:1EA): 0.6. Melting point: >320 °C (decomp.). ^1^H–NMR (500 MHz, DMSO-d_6_) *δ*: 12.51 (brs, 1H), 8.07 (d, *J* = 2.0 Hz, 1H), 8.03(d, *J* = 9.6 Hz, 1H), 7.95 (dd, *J* = 8.7, 2.0 Hz, 1H), 7.63 (d, *J* = 16.0 Hz, 1H), 7.43 (d, *J* = 8.6 Hz, 1H), 6.58 (d, *J* = 16.0 Hz, 1H), 6.54 (d, *J* = 9.4 Hz, 1H). ^13^C–NMR (125 MHz, DMSO-d_6_) δ: 167.4, 159.7, 154.4, 144.0, 142.3, 131.5, 130.7, 128.3, 119.90, 117.0, 116.9, 119.1., LC-MS (ESI, *m/z*): [M + H]^+^ = 217.

#### 3.1.4. General Method for the Synthesis of Acrylic and Oxo Amides 4–12 [47]

**Figure 3:**
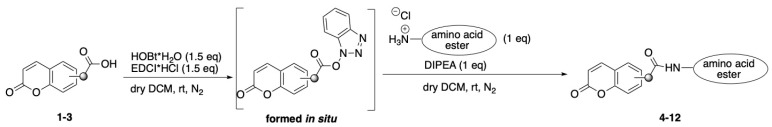
Synthesis of compounds **4**–**12**.

In a round-bottomed flask were subsequently added the following: dry dichloromethane (10 mL), the appropriate acid (0.68 mmol, 1 eq), 1-hydroxybenzotriazole monohydrate (HOBt*H_2_O) (136.5 mg, 1.01 mmol, 1.5 eq), and 1-ethyl-3-(3-dimethylaminopropyl)carbodiimide hydrochloride (EDCI*HCl) (1936 mg, 1.01 mmol, 1.5 eq). The mixture was stirred at room temperature under a nitrogen atmosphere. Upon consumption of the acid, equimolar quantities of the corresponding amino acid ester hydrochloride salt (0.68 mmol, 1 eq) and N,N-diisopropylethylamine (DIPEA) (118.5 μL, 0.68 mmol, 1 eq) were added. The final mixture was stirred overnight at room temperature under a nitrogen atmosphere. Upon completion (TLC monitoring), the reaction mixture was transferred to a separation funnel and washed with brine solution (3 × 10 mL). The organic layer was collected, dried over MgSO_4_, and evaporated to dryness. The residue was either recrystallized from the appropriate solvent or purified using column chromatography, as stated in each case. The final compounds were isolated in moderate to excellent yields (Figure 3).

##### Ethyl (E)-(3-(2-Oxo-2H-Chromen-6-yl)Acryloyl)Glycinate (4)

White solid. Yield: 58%. R_f_ (1PS:3EA): 0.4. Melting point: 188–190 °C (this compound was recrystallized from petroleum spirit). IR (KBr, cm^−1^): 3305.3, 1703.4, 1661.5. ^1^H–NMR (500 MHz, DMSO-d_6_) *δ*: 8.57 (t, *J* = 5.8 Hz, 1H), 8.08 (d, *J* = 9.6 Hz, 1H), 7.94 (d, *J* = 1.2 Hz, 1H), 7.84 (dd, *J* = 8.6, 1.5 Hz, 1H), 7.51 (d, *J* = 15.8 Hz, 1H), 7.45 (d, *J* = 8.6 Hz, 1H), 6.74 (d, *J* = 15.8 Hz, 1H), 6.54 (d, *J* = 9.6 Hz, 1H), 4.12 (q, *J* = 7.1 Hz, 2H), 3.97 (d, *J* = 5.9 Hz, 2H), 1.21 (t, *J* = 7.1 Hz, 3H). ^13^C–NMR (125 MHz, DMSO-d_6_) *δ*: 169.9, 165.2, 159.68, 154.1, 144.1, 137.9, 131.2, 130.7, 127.8, 122.0, 119.1, 117.1, 116.8, 60.5, 40.9, 14.1. HRMS (ESI, *m/z*), (M.W.: 301): [M + H]^+^ theoretically for C_16_H_16_NO_5_: 302.1028, found: 302.1022.

##### Methyl (E)-4-(3-(2-Oxo-2H-Chromen-6-yl)Acrylamido)Butanoate (5)

White solid. Yield: 100%. R_f_ (1PS:3EA): 0.4. Melting point: 140–142 °C (this compound was recrystallized from petroleum spirit). IR (KBr, cm^−1^): 3300.4, 1759.4, 1730.3, 1654.1. ^1^H–NMR (500 MHz, DMSO-d_6_) *δ*: 8.18 (t, *J* = 5.7 Hz, 1H), 8.07 (d, *J* = 9.6 Hz, 1H), 7.90 (d, *J* = 2.1 Hz, 1H), 7.81 (dd, *J* = 8.7, 2.1 Hz, 1H), 7.46 (d, *J* = 13.3 Hz, 1H), 7.44 (d, *J* = 6.1 Hz, 1H), 6.62 (d, *J* = 15.8 Hz, 1H), 6.54 (d, *J* = 9.6 Hz, 1H), 3.59 (s, 3H), 3.20 (dd, *J* = 12.7, 6.8 Hz, 2H), 2.36 (t, *J* = 7.4 Hz, 2H), 1.72 (quint, *J* = 7.2 Hz, 2H). ^13^C–NMR (125 MHz, DMSO-d6) δ: 173.1, 164.7, 159.7, 153.9, 144.0, 137.0, 131.4, 130.6, 127.6, 122.8, 119.0, 117.0, 116.8, 51.3, 38.0, 30.7, 24.5. HRMS (ESI, *m/z*), (M.W.: 315): [M + Na]^+^ theoretically for C_17_H_17_NO_5_Na: 338.1005, found: 338.0997.

##### (-)-Dimethyl (E)-(3-(2-Oxo-2H-Chromen-6-yl)Acryloyl)-L-Glutamate (6)

Off-white solid. Yield: 66%. R_f_ (10%*v/v* MeOH:CH_2_Cl_2_): 0.4. Melting point: 85–87 °C (the compound was purified by column chromatography using 10% *v/v* MeOH:CH_2_Cl_2_ as eluent). ${\left[a\right]}_{589}^{25}=\;-13.3$° ($9\times 10^{-3}\frac{g}{mL}$ in MeOH). IR (KBr, cm^−1^): 3378.7, 1734.0, 1718.4, 1670.7, 1623.3 ^1^H–NMR (500 MHz, DMSO-d_6_): *δ* 8.58 (d, *J* = 7.5 Hz, 1H), 8.07 (d, *J* = 9.6 Hz, 1H), 7.92 (d, *J* = 1.7 Hz, 1H), 7.83 (dd, *J* = 8.6, 1.9 Hz, 1H), 7.50 (d, *J* = 15.8 Hz, 1H), 7.45 (d, *J* = 8.6 Hz, 1H), 6.71 (d, *J* = 15.8 Hz, 1H), 6.54 (d, *J* = 9.6 Hz, 1H), 4.42 (td, *J* = 8.3, 5.7 Hz, 1H), 3.65 (s, 3H), 3.59 (s, 3H), 2.44–2.41 (m, 2H), 2.09–2.02 (m, 1H), 1.94–1.87 (m, 1H). ^13^C–NMR (125 MHz, DMSO-d_6_): δ 172.5, 172.1, 165.0, 159.7, 154.1, 144.0, 138.1, 131.2, 130.7, 127.9, 121.9, 119.1, 117.1, 116.8, 68.0, 51.4, 29.7, 26.2. HRMS (ESI, *m/z*), (M.W.: 373): [M + H]^+^ theoretically for C_19_H_20_NO_7_: 374.1234, found: 374.1240.

##### Ethyl (2-((2-Oxo-2H-Chromen-6-yl)Oxy)Acetyl)Glycinate (7)

White solid. Yield: 39%. R_f_ (10% *v/v* MeOH:CHCl_3_) = 0.4. Melting point = 119–121 °C (this compound was purified by column chromatography using 5% *v/v* MeOH:CHCl_3_ as eluent). IR (KBr, cm^−1^): 3382.0, 1748.8, 1713.5, 1678.3. ^1^H–NMR (500 MHz, CDCl_3_) *δ* 7.66 (d, *J* = 9.5 Hz, 1H), 7.31 (d, *J* = 9.1 Hz, 1H), 7.18 (dd, *J* = 9.1, 3.0 Hz, 1H), 7.05 (s, 1H), 6.99 (d, *J* = 2.9 Hz, 1H), 6.46 (d, *J* = 9.6 Hz, 1H), 4.58 (s, 2H), 4.24 (q, *J* = 7.1 Hz, 2H), 4.13 (d, *J* = 5.4 Hz, 2H), 1.30 (t, *J* = 7.2 Hz, 3H). ^13^C–NMR (125 MHz, DMSO-d_6_): δ 169.6, 168.0, 160.1, 154.0, 148.3, 144.0, 120.0, 119.1, 117.4, 116.8, 112.1, 95.7, 67.3, 60.5, 14.1. HRMS (ESI, *m/z*), (M.W.: 305): [M + Na]^+^ theoretically for C_15_H_15_NO_6_Na: 328.0797, found: 328.0789.

##### Methyl 4-(2-((2-Oxo-2H-Chromen-6-yl)Oxy)Acetamido)Butanoate (8)

White solid. Yield: 66%. R_f_ (10% *v/v* MeOH:CH_2_Cl_2_): 0.7. Melting point = 125–127 °C (the compound was purified by column chromatography using 10% *v/v* MeOH:CH_2_Cl_2_ as eluent). IR (KBr, cm^−1^): 3375.7, 1742.5, 1717.4, 1668.4. ^1^H–NMR (400 MHz, CDCl_3_): *δ* 7.66 (d, *J* = 9.6 Hz, 1H), 7.31 (d, *J* = 9.1 Hz, 1H), 7.17 (dd, *J* = 9.1, 2.9 Hz, 1H), 6.95 (d, *J* = 2.8 Hz, 1H), 6.81 (s, 1H), 6.46 (d, *J* = 9.6 Hz, 1H), 4.51 (s, 2H), 3.67 (s, 3H), 3.41 (q, *J* = 6.6 Hz, 2H), 2.38 (t, *J* = 7.1 Hz, 2H), 1.90 (quint, *J* = 7.0 Hz, 2H). ^13^C–NMR (100 MHz, CDCl_3_): *δ* 173.8, 167.8, 160.9, 153.8, 149.4, 143.0, 120.0, 119.5, 118.4, 117.7, 111.3, 68.0, 51.9, 38.7, 31.6, 24.6. HRMS (ESI, *m/z*), (M.W.: 319): [M + Na]^+^ theoretically for C_16_H_17_NO_6_Na: 342.0950, found: 342.0945.

##### (-)-Dimethyl (2-((2-Oxo-2H-Chromen-6-yl)Oxy)Acetyl)-L-Glutamate (9)

White solid. Yield: 71%. R_f_ (10% *v/v* MeOH:CH_2_Cl_2_): 0.8. Melting point: 113–115 °C (the compound was purified by column chromatography using 10% *v/v* MeOH:CH_2_Cl_2_ as eluent). ${\left[a\right]}_{589}^{25}=\;-11$° ($9\times 10^{-3}\frac{g}{mL}$ in MeOH). IR(KBr, cm^−1^): 3349.0, 1751.9, 1702.4, 1674.9. ^1^H–NMR (400 MHz, CDCl_3_): *δ* 7.65 (d, *J* = 9.6 Hz, 1H), 7.31 (d, *J* = 8.1 Hz, 1H), 7.28 (d, *J* = 9.1 Hz, 1H), 7.18 (dd, *J* = 9.0, 2.8 Hz, 1H), 6.98 (d, *J* = 2.8 Hz, 1H), 6.43 (d, *J* = 9.5 Hz, 1H), 4.68 (td, *J* = 7.9, 5.1 Hz, 1H), 4.53 (d, *J* = 2.1 Hz, 2H), 3.75 (s, 3H), 3.63 (s, 3H), 2.45–2.35 (m, 2H), 2.32–2.21 (m, 1H), 2.10–2.00 (m, 1H). ^13^C–NMR (100 MHz, CDCl_3_): *δ* 173.3, 171.8, 167.8, 160.7, 153.7, 149.4, 143.00, 120.0, 119.5, 118.4, 117.6, 111.6, 68.0, 52.8, 52.0, 51.5, 30.1, 27.1. HRMS (ESI, *m/z*), (M.W.: 377): [M + H]^+^ theoretically for C_18_H_20_NO_8_: 378.1184, found: 378.1184.

##### Ethyl (2-((2-Oxo-2H-Chromen-7-yl)Oxy)Acetyl)Glycinate (10)

White solid. Yield: 45% R_f_ (10% *v/v* MeOH:CHCl_3_): 0.4. Melting point = 128–130 °C (the compound was purified by column chromatography using 5% *v/v* MeOH:CHCl_3_ as eluent). IR (KBr, cm^−1^): 3391.2, 1748.2, 1717.4, 1673.8, ^1^H–NMR (500 MHz, CDCl_3_) *δ* 7.63 (d, *J* = 9.5 Hz, 1H), 7.41 (d, *J* = 8.6 Hz, 1H), 7.13 (s, 1H), 6.88 (dd, *J* = 8.6, 2.4 Hz, 1H), 6.83 (d, *J* = 2.3 Hz, 1H), 6.26 (d, *J* = 9.5 Hz, 1H), 4.57 (s, 2H), 4.21 (d, *J* = 7.1 Hz, 2H), 4.11 (d, *J* = 5.5 Hz, 2H), 1.26 (t, *J* = 7.1 Hz, 3H). ^13^C–NMR (125 MHz, CDCl_3_) δ 169.4, 167.4, 160.8, 160.1, 155.7, 143.2, 129.3, 114.2, 113.8, 112.3, 102.5, 67.5, 61.8, 41.0, 14.2. HRMS (ESI, *m/z*), (M.W.: 305): [M + H]^+^ theoretically for C_15_H_16_NO_6_: 306.0977, found: 306.0975.

##### Methyl 4-(2-((2-Oxo-2H-Chromen-7-yl)Oxy)Acetamido)Butanoate (11)

White solid. Yield: 75%. R_f_ (10% *v/v* MeOH:CH_2_Cl_2_): 0.7. Melting point: 115–118 °C (the compound was purified by column chromatography using 10% *v/v* MeOH:CH_2_Cl_2_ as eluent). IR (KBr, cm^−1^): 3386.0, 1732.0, 1708.9, 1669.4. ^1^H–NMR (400 MHz, CDCl_3_): *δ* 7.65 (d, *J* = 9.5 Hz, 1H), 7.44 (d, *J* = 8.4 Hz, 1H), 6.91 (d, *J* = 2.3 Hz, 1H), 6.88 (s, 1H), 6.84 (s, 1H), 6.30 (d, *J* = 9.5 Hz, 1H), 4.54 (s, 2H), 3.68 (s, 3H), 3.42 (dd, *J* = 12.9, 6.6 Hz, 2H), 2.39 (t, *J* = 7.0 Hz, 2H), 1.91 (quint, *J* = 6.9 Hz, 2H). ^13^C–NMR (100 MHz, CDCl_3_): *δ* 173.7, 167.2, 160.8, 160.1, 155.5, 143.2, 129.2, 113.9, 113.5, 112.1, 102.4, 67.4, 51.7, 38.6, 31.4, 24.4. HRMS (ESI, *m/z*), (M.W.: 319): [M + H]^+^ theoretically for C_16_H_18_NO_6_: 320.1127, found: 320.1131.

##### (-)-Dimethyl (2-((2-Oxo-2H-Chromen-7-yl)Oxy)Acetyl)-L-Glutamate (12)

White solid. Yield: 54%. R_f_ (10% *v/v* MeOH:CH_2_Cl_2_): 0.8. Melting point: 119–121 °C (the compound was purified by column chromatography using 10% *v/v* MeOH:CH_2_Cl_2_ as eluent). ${\left[a\right]}_{589}^{25}=\;-8$ $(11\times 10^{-3}\frac{g}{mL}$ in MeOH) deg × mL × g^−1^ × dm^−1^. IR (KBr, cm^−1^): 3321.9, 1745.4, 1711.8, 1689.5, 1675.5 ^1^H–NMR (400 MHz, CDCl_3_): *δ* 7.61 (d, *J* = 9.5 Hz, 1H), 7.39 (d, *J* = 8.4 Hz, 2H), 6.88 (dd, *J* = 8.6, 1.7 Hz, 1H), 6.83 (s, 1H), 6.23 (d, *J* = 9.5 Hz, 1H), 4.64 (dd, *J* = 12.9, 7.8 Hz, 1H), 4.53 (s, 2H), 3.71 (s, 3H), 3.61 (s, 3H), 2.42–2.32 (m, 2H), 2.27–2.20 (m, 1H), 2.08–1.99 (m, 1H). ^13^C–NMR (100 MHz, CDCl_3_): *δ* 173.3, 171.7, 167.3, 160.8, 160.1, 155.6, 143.2, 129.2, 114.0, 113.7, 112.4, 102.4, 67.3, 52.7, 51.9, 51.5, 30.0, 26.8. HRMS (ESI, *m/z*), (M.W.: 377): [M + H]^+^ theoretically for C_18_H_20_NO_8_: 378.1184, found: 378.1184.

#### 3.1.5. Synthesis of 6-Nitro-2H-Chromen-2-One (13) [67]

**Figure 4:**
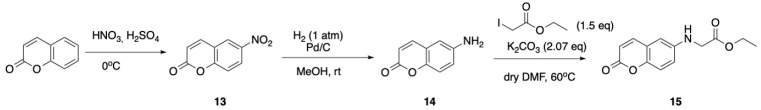
Synthesis of compounds **13** to **15**.

In a round-bottomed flask equipped with a magnetic stirrer, 3 mL of concentrated H_2_SO_4_ was added. The solution was cooled to 0 °C, and then 1.46 g (10 mmol, 1 eq) of 2*H*-chromen-2-one was dissolved. Upon dissolution, a mixture of concentrated (98.3%) H_2_SO_4_ and concentrated (68%) HNO_3_ (1 mL:1 mL) was added dropwise. The reaction was stirred at 0 °C for 2 h. Upon consumption of the starting material, the reaction mixture was poured onto a mixture of water and ice, and a white solid formed. The solid was filtered off, washed with water, dried, and recrystallized from ethyl acetate to a white solid (Figure 4).

##### 6-Nitro-2H-Chromen-2-One (13)

White solid. Yield: 65%. Melting point: 178–180 °C (ref: 179–181 °C [68]. ^1^H–NMR (500 MHz, CDCl_3_) *δ* 8.44 (d, *J* = 2.5 Hz, 1H), 8.40 (dd, *J* = 9.0, 2.6 Hz, 1H), 7.80 (d, *J* = 9.7 Hz, 1H), 7.47 (d, *J* = 9.0 Hz, 1H), 6.59 (d, *J* = 9.6 Hz, 1H). LC-MS (ESI, *m/z*): [M + H]^+^ = 192.

#### 3.1.6. Synthesis of 6-Amino-2H-Chromen-2-One (14) [69]

Yellow solid. Yield: 100%. Melting point: 142–144 °C (ref: 142–146 °C [70]). ^1^H–NMR (500 MHz, DMSO-d_6_): *δ* 7.88 (d, *J* = 9.5 Hz, 1H), 7.10 (d, *J* = 8.8 Hz, 1H), 6.84 (dd, *J* = 8.8, 2.8 Hz, 1H), 6.74 (d, *J* = 2.6 Hz, 1H), 6.35 (d, *J* = 9.6 Hz, 1H), 5.24 (s, 2H). LC − MS (ESI, *m/z*): [M + H]^+^ = 162.

#### 3.1.7. Synthesis of Ethyl (2-Oxo-2H-Chromen-6-yl)Glycinate (15) [47]

In a round-bottomed flask equipped with a magnetic stirrer, 7 mL of dry dimethylformamide (DMF) was added. Then, 483.5 mg (3 mmol, 1 mmol) of 6-amino-2*H*-chromen-2-one and 858 mg (6.21 mmol, 2.07 eq) of potassium carbonate were added. The mixture was stirred for 15 min at room temperature and then 533 μL (4.5 mmol, 1.5 eq) of ethyl iodoacetate was added dropwise. The mixture was heated at 60 °C for 30 min under argon atmosphere. Upon completion of the reaction (TLC monitoring), the solution was left to cool to room temperature and was quenched with water. The yellow solid formed was filtrated, washed with water, and kept at 4 °C (due to degradation at higher temperatures, as verified by TLC) and was used without any further purification.

##### Ethyl (2-Oxo-2H-Chromen-6-yl)Glycinate (15)

Yellow solid. Yield: 41%. R_f_ (1PS:1EA): 0.4. Melting point: 111–112 °C (decomp.). ^1^H–NMR (500 MHz, CDCl_3_): *δ* 7.60 (d, *J* = 9.5 Hz, 1H), 7.20 (d, *J* = 8.9 Hz, 1H), 6.87 (dd, *J* = 8.8, 2.6 Hz, 1H), 6.60 (d, *J* = 2.3 Hz, 1H), 6.39 (d, *J* = 9.5 Hz, 1H), 4.26 (q, *J* = 7.1 Hz, 2H), 3.93 (s, 2H), 1.31 (t, *J* = 7.1 Hz, 3H). ^13^C–NMR (125 MHz, CDCl_3_): *δ* 170.9, 161.3, 147.4, 144.0, 143.4, 119.5, 118.5, 117.9, 117.3, 108.9, 61.7, 46.2, 13.4. HRMS (ESI, m/z), (M.W. 247:) [M + H]^+^ theoretically for C_13_H_14_NO_4_: 248.0917, found: 248.0914.

### 3.2. Biology

#### 3.2.1. Instrumentation

For the Ultraviolet–Visible (UV–Vis) experiments, a Perkin-Elmer UV/Vis Spectrometer Lamda 20 and a Shimadzu UV–Vis Spectrophotometer PharmaSpec UV-1700 apparatus were implemented.

#### 3.2.2. Biological Reagents

Biological assays were conducted using reagents, including soybean lipoxygenase, sodium linoleate, 2,2′-azobis(2-amidinopropane) dihydrochloride (AAPH), Trolox, nordihydroguaiaretic acid (NDGA), N,N,N′,N′-tetramethyl-p-phenylenediamine (TMPD), heme, phosphate buffer (pH 7.4), and Tris(hydroxymethyl)aminomethane (pH 9.0), all purchased from Merck and Sigma-Aldrich (Darmasdat, Germany), and ovine cyclooxygenase-2 was purchased from Cayman Chemical Ann Arbor, MI, USA.

#### 3.2.3. Biological Assays

The biological experiments were replicated three or four times. Values are means ± SD of the applied three or four different determinations. Means within each column differ significantly (*p* < 0.05). In all cases, statistical significances against controls were performed by the Student’s *t*-test. NDGA and indomethacin were used as references and as positive controls. Identical experimental conditions (enzyme concentration, substrate concentration, and incubation time) were used for both references and tested compounds to ensure the standardization of IC_50_ value comparisons.

##### *In Vitro* AAPH-Induced Sodium Linoleate Peroxidation Inhibition Assay

Compounds were tested for the inhibition of lipid peroxidation of sodium linoleate, induced by AAPH at 37 °C. Detailed experimental information is described in the supplementary data file.

##### *In Vitro* Soybean Lipoxygenase Inhibitory Activity

The designed molecules were evaluated as soybean lipoxygenase inhibitors [71]. Experimental details are given in the supplementary section.

##### Ovine Cyclooxygenase 2 (COX-2) Inhibitory Activity

Compounds were tested as ovine cyclooxygenase 2 inhibitors [71]. The methodology is described in the Supplementary section.

### 3.3. In Silico Software

For the ADMET calculations, the following free platforms were used: Molinspiration (https://www.molinspiration.com, accessed on 14 June 2021), SwissADME (www.swissadme.ch/, accessed on 27 July 2021), PreADMET (https://preadmet.qsarhub.com/adme/, accessed on 15 May 2021), Molsoft (https://www.molsoft.com/mprop/, accessed on 17 August 2021) GLORYx (https://nerdd.univie.ac.at/gloryx/, accessed on 6 May 2021), CypRules (https://cyprules.cmdm.tw/, accessed on 17 August 2021), and LiverTox Workspace (https://pharminfo.univie.ac.at/people/melanie-grandits/details/pure/fb6f0da1-a9ab-4883-a818-601238800c27/show/publ/Pure/, accessed on 14 September 2021).

## 4. Conclusions

The designed and synthesized coumarin acid and amino acid hybrids presented a multitarget activity against inflammatory targets. The compounds were designed to combine the promising biological activity of the coumarin moiety against LOX and COX enzymes, as well as the neuromodulatory activity of glycine, GABA, and glutamate amino acids in a single molecule.

The synthesis of the desired compounds (**4–12**) proceeded via a one-pot reaction from the corresponding acid using EDCI/HOBt-mediated amide synthesis. This protocol was selected due to its facile implementation, easy workup, and small probability of epimerization of the glutamate moiety. The final compounds were isolated in medium to excellent yields (39–100%). Compound **15** was synthesized in a medium overall yield (27%) using a three-step procedure from 2H-chromen-2-one.

Antioxidant activity was evaluated using the AAPH-induced sodium linoleate peroxidation inhibition assay. The results clearly show that the 6-oxo-linker yields more active compounds, mainly due to the atom’s more efficient radical-stabilizing capability in this position. The inhibition assay against soybean lipoxygenase highlighted compound **4** as the most active agent, with an IC_50_ value of 58 μΜ. In this protocol, the acrylic compounds **4–6** were more active than the 6-oxo and 7-oxo hybrids **7–12** and the amine derivative **15**. This may be due to their structural resemblance to cinnamic acid derivatives, a known class of LOX inhibitors. The inhibitory activity against ovine COX-2 indicated compounds **4** and **5** as the most active agents, with IC_50_ values of 55 μM each.

Compound **4** is a glycinate hybrid. Glycine, as an essential amino acid, acts as an anti-inflammatory agent that inhibits the expression of COX-2 (cyclooxygenase-2), reduces the production of inflammatory prostaglandins, and plays a significant role in modulating the lipoxygenase (LOX) pathway, primarily acting as a stress-response regulator. Acrylic acid did not exhibit COX-2 inhibition, while its LOX inhibition was moderate. Studies [72] on glycinate samples show that it downregulates COX-2 protein levels in activated macrophages. It is shown to significantly decrease mRNA and protein expression of COX-2 in various tissues, including the kidneys and blood vessels. This reduction helps lower the production of pro-inflammatory prostanoids, such as PGE2 and kidney tissues, suggesting a potential role in managing inflammatory diseases. Glycine exerts anti-inflammatory effects by inhibiting inflammatory cytokine activity, such as TNF-α and peroxide secretion, in mouse alveolar macrophages stimulated with LPS. It also suppresses expression of inflammatory factors, such as IL-6, in the 3T3-L1 cell line and monocytes [73,74,75]. Thus, the significant response of hybrid 4 is mechanistically related to the presence of the glycinate moiety. Since only preliminary screening results are available, we believe that it is worth furthering investigations of **4** in the near future, e.g., in cellular inflammation models or cytokine assays.

Once again, the acrylic core is the most privileged scaffold used as a linker, as it elevated activity (e.g., compound **4** IC_50_ = 55 μM versus compound **15** with 29% inhibition). From the biological experiments, compounds **4, 9**, and **15** are depicted as pleiotropic molecules due to their multitarget activity (for compound **4**: IC_50_ for SLOX-1 = 58 μM and for COX-2 = 55 μM; for compound **9:** IC_50_ value for COX-2 = 60 μM, 59% AAPH-induced inhibition; for compound **15:** IC_50_ value for COX–2 = 70.5 μM, 93% AAPH-induced inhibition).

The *in silico* drug-likeness and pharmacokinetic profile showed that they can all be well absorbed after per os administration and have a medium protein binding profile. None of the designed compounds seems to penetrate the BBB via passive diffusion, although their amino-acid moiety can facilitate their active-transport penetration. Once they penetrate the BBB, none of the compounds can exit because none showed a Pgp substrate probability. The only exception is compound **9,** which, on the PreADMET column, seems to be a substrate of the Pgp protein, while on the SwissADME platform, it is not considered a substrate. This can be attributed to the different training sets for each platform. Pharmacokinetically, all compounds show good metabolic and CYP inhibitory profiles since none of the compounds inhibit CYP enzymes or show any toxic metabolic profile. On the contrary, they all show a high probability of exerting hepatotoxic activities since they can interact with a plethora of transporters. Considering that these findings suggest an initial *in silico* warning for their toxicity, structural modifications will be studied, and experimental hepatotoxicity data will be obtained in order to design more active agents against LOX and COX-2, with fewer hepatotoxic properties.

Regardless of their hepatotoxic probability, all compounds showed very promising drug-likeness. Their properties were quantified by calculating their QED scores. All compounds exerted a QED score greater than 0.5 and were very close (or even exceeded) to the QED scores of the reference compounds used in the biological experiments. Hybrids **4**, **5**, and **9** are the most potent, multitarget derivatives and dual inhibitors of COX-2 and LOX. Compound **4** is presented to have favorable drug-likeness and predicted anti-inflammatory activity, inhibiting carrageenin foot paw edema at 44%. Compound **15** is also a potent antioxidant and COX-2 inhibitory agent. This molecule will be used as a lead compound for further investigation and structural modifications.

## Figures and Tables

**Table 1:** Biochemical evaluation of the synthesized compounds.

Compound	Structure	% Lipid Peroxidation Inhib. (±SD)	%SLOX Inhib. (100 μM) or IC_50_ Value µM (±SD)	%COX-2 Inhib. (100 μM) or IC_50_ Value µM (±SD)
**1**	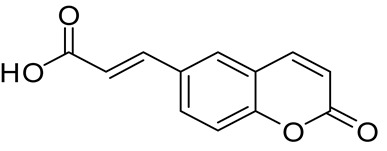	n.a.	31% (±0.5)	n.a.
**4**	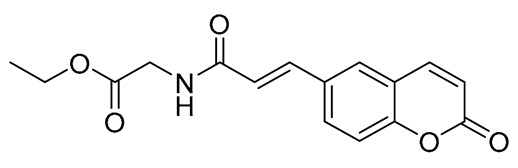	37% (±0.9)	58 μΜ (±0.4)	55 μΜ (±1.3)
**5**	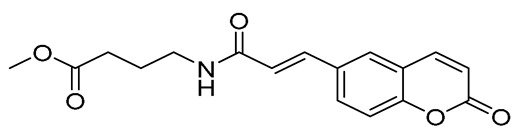	n.a.	41% (±1.6)	55 μΜ (±0.8)
**6**	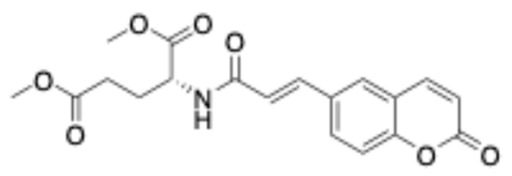	23% (±1.1)	32% (±1.3)	n.a.
**2**	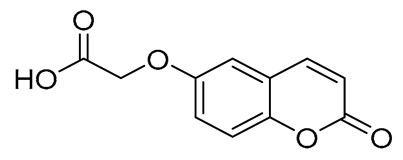	45% (±1.9)	100 μΜ [47]	n.a.
**7**	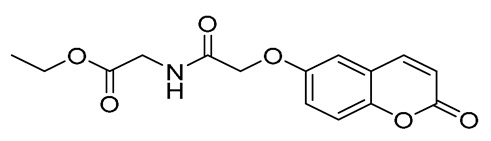	39% (±1.2)	n.a.	n.a.
**8**	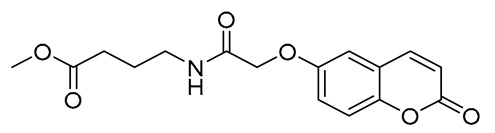	32% (±0.6)	n.a.	44% (±1.4)
**9**	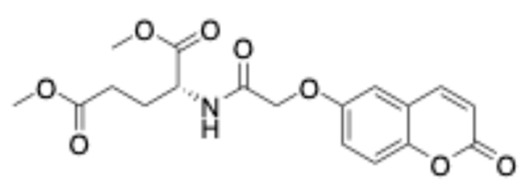	59% (±2.3)	100 μΜ (±3.2)	60 μΜ (±1.8)
**3**	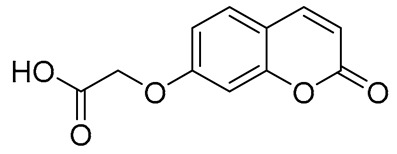	1% (±0.02)	100 μΜ [47]	n.a.
**10**	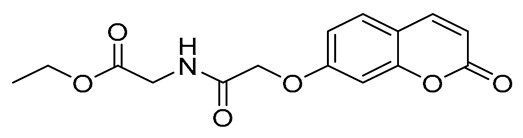	28% (±0.8)	n.a.	17% (±0.2)
**11**	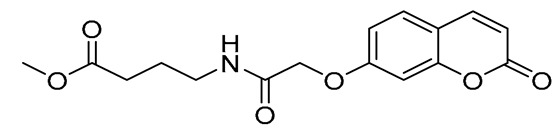	13% (±0.1)	n.a.	n.a.
**12**	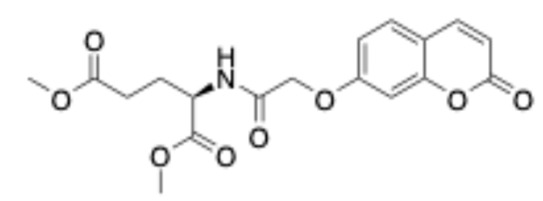	22% (±0.4)	n.a.	n.a.
**15**	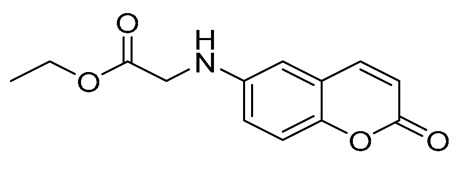	93% (±2.1)	29% (±0.5)	70.5 μΜ (±1.9)
**Trolox**	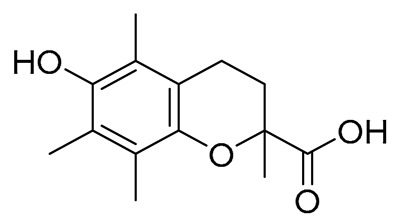	92% (±2.8)	n.t.	n.t.
**NDGA**	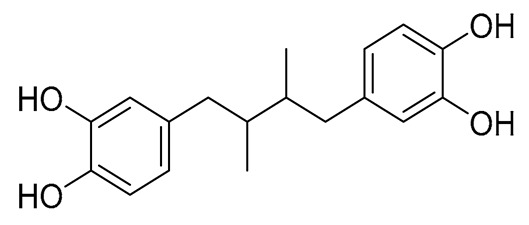	n.t.	0.45 μΜ (±0.013)	n.t.
**Indomethacin**	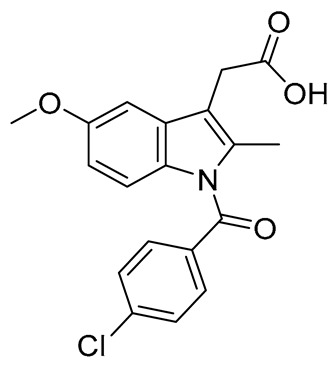	n.t.	n.t.	1.12 μΜ (±0.4)

Values are means ± SD of three or four different determinations; na, no activity under the experimental conditions. Means within each column differ significantly (*p* < 0.05). In all cases, statistical significances against controls were performed by Student’s *t*-test; nt = not tested: this compound was not studied in this experiment.

## Data Availability

The produced compounds are found in the Laboratory of Pharmaceutical Chemistry, and they can be provided by the authors after asking for them.

## Supplementary Materials

The following supporting information can be downloaded at: https://www.mdpi.com/article/10.3390/ijms27104443/s1.

## Abbreviations

The following abbreviations are used in this manuscript:

AD	Alzheimer’s Disease
ADMET	Absorption, Distribution, Metabolism, Excretion, Toxicity
CNS	Central Nervous System
COX	Cyclooxygenase
CPE	Carrageenin-induced rat paw edema
DMSO	Dimethyl sulfoxide
EA	Ethyl acetate
GABA	gamma aminobutyric acid
FT–IR	Fourier-Transformation Infrared Spectroscopy
LOX	Lipoxygenase
MeOH	Methanol
PGG_2_	Prostaglandin G_2_
PGH_2_	Prostaglandin H_2_
PLA_2_	Phospholipase A_2_
PS	Petroleum Spirit
PTGS2	Prostaglandin-endoperoxide synthase 2
PUFA	Poly-Unsaturated Fatty Acid
ROS	Reactive Oxygen Species
RNS	Reactive Nitrogen Species
TLC	Thin Layer Chromatography
Tris-HCl	Tris(hydroxymethyl)aminomethane hydrochloride
TXA_2_	Thromboxane A_2_
VEGFR-2	Vascular Endothelial Growth Factor Receptor 2
QED	Quantitative Estimate of Druglikeness
