# Supplementary material for: Coumarin–Amino Acid Hybrids Used as Possible Multifactorial Anti-Inflammatory Agents
Source: Int J Mol Sci. 2026 May 15;27(10):4443. doi: 10.3390/ijms27104443 (PMC13207833; doi:10.3390/ijms27104443)
Supplement: Supplementary file 1 [file ijms-27-04443-s001.zip › ijms-4269131-supplementary.pdf]

International Journal of *Molecular Sciences*

MDPI

Article

Coumarin – amino acids hybrids, as possible multifactorial anti-inflammatory agents

Ioannis Fotopoulos * and Dimitra Hadjipavlou-Litina

Laboratory of Pharmaceutical Chemistry, School of Pharmacy, Faculty of Health Sciences, Aristotle University of Thessaloniki, Thessaloniki, 54124, Greece

* Correspondence authors: ioanfoto@pharm.auth.gr; hadjipav@pharm.auth.gr

Supplementary Material

1. Biological protocols

For the biological experiments, stock 10 mM solutions of the compounds (see Figure S1), in DMSO, were prepared. Solutions of lower concentrations were prepared by diluting the stock solutions accordingly. The biological experiments were performed in triplicate, and the results are the mean of three experiments. The standard deviation is less than 10%.

1.1. AAPH-induced peroxidation inhibition assay.

In a 1 mL quartz cuvette, at 37°C, 930 μL of phosphate buffer solution was added (0.05 M pH = 7.4). Subsequently, 10 μL of 16 mM aqueous sodium linoleate solution, 10 μL of 100 μM compound solution and 50 μL of 40 mM aqueous AAPH solution are added. The reaction was started upon addition of AAPH, due to the formation of the 13-hydroperoxy-linolate radical. The absorbance was recorded at 234 nm. DMSO is used as a negative control. Trolox is used as a reference compound. The results are given in Table 1 in the main manuscript.

1.2. Soybean lipoxygenase inhibitory activity.

A protocol developed by our group was used [71]. In a 1 mL quartz cuvette, at room temperature, the studied compounds were incubated with sodium linoleate (0.1 mM final concentration) and the enzyme (1/9 x 10^{-4} w/v in saline, 0.2 mL volume). The reaction was monitored at 234 nm due to the formation of the 13-

 https://doi.org/10.3390/ijms27104443

hydroperoxy-linoleate radical. Nor-dihydroguaeretic acid (NDGA) was used as positive control (IC_{50} = 0.45 μM). The results are given in Table 1 in the main manuscript.

1.3. Ovine cyclooxygenase 2 (COX-2) inhibitory activity.

The in vitro COX-2 inhibitory activity was assessed using arachidonic acid (AA) as a substrate and N,N,N',N'-tetramethyl-p-phenylenediamine (TMPD) as a co-substrate in a Tris-HCl buffer (pH 8.5), following the reported method [71]. To the test tube, 10 μL of the compound dissolved in DMSO was added to a mixture containing 730 μL of Tris-HCl buffer (pH 8.5), 50 μL of heme, 10 μL of COX-2 enzyme, and 100 μL of TMPD. The absorbance was measured at 590 nm. Subsequently, 100 μL of AA was added, followed by a second 5-minute incubation at 37°C with vigorous shaking, and the absorbance was measured again at 611nm. Different concentrations (ranging from 100 μM to 1 μM) were tested to determine IC_{50} values. A blank determination was conducted as a negative control. Results were compared against the standard inhibitor Indomethacin. The results are given in Table 1 in the main manuscript.

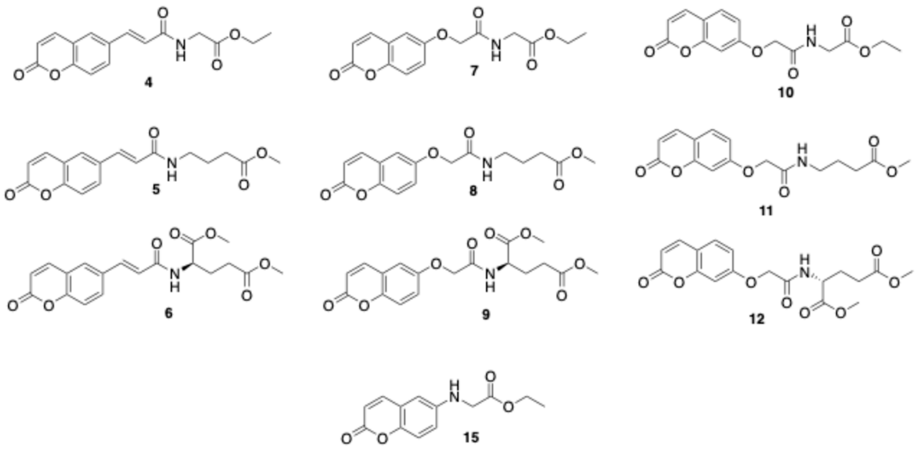

Figure S 1 - Structures of the designed compounds.

2. Computational Data

2.1. Physicochemical Data using the Molinspiration platform.

https://doi.org/10.3390/ijms27104443

In the following Table S1, the retrieved data from the Molinspiration platform are depicted. The abbreviations used are as follows: **miLogP** = predicted lipophilicity, **TPSA**: Molecular Polar Surface Area (Å^2), **MW** = molecular weight, **N_{ON}** = number of oxygen and nitrogen atoms (hydrogen bond acceptors), **N_{OHNH}** = number of alcohol and amine groups (hydrogen bond donors), **N_{viol}** = number of "rule-of-5" violations, **Rot** = rotatable bonds, **MV** = molecular volume (Å^3).

Table S1 - Physicochemical data retrieved from the Molinspiration platform.

Compound	miLogP	TPSA	MW	N_{ON}	N_{OHNH}	N_{viol}	Rot	MV
**4**	2.06	85.61	301.30	6	1	0	6	265.52
**5**	1.92	85.61	315.32	6	1	0	7	282.32
**6**	1.77	111.92	373.36	8	1	0	9	326.88
**7**	1.39	94.85	305.29	7	1	0	7	263.89
**8**	1.25	94.85	319.31	7	1	0	8	280.69
**9**	1.10	121.15	377.35	9	1	0	10	325.25
**10**	1.39	94.85	305.29	7	1	0	7	263.89
**11**	1.25	94.85	319.31	7	1	0	8	280.69
**12**	1.10	121.15	377.35	9	1	0	10	325.25
**15**	2.09	68.54	247.25	5	1	0	5	219.12

https://doi.org/10.3390/ijms27104443

2.2. Blood – Brain barrier calculations using SwissADME, PreADMET and MolSoft tools.

In the following Table S2, the blood – barrier penetration calculations using the SwissADME, PreADMET and MolSoft tools are shown.

Table S2 - Blood - brain barrier calculations using the SwissADME, PreADMET and MolSoft tools.

Compound	SwissADME		PreADMET		MolSoft
	Blood-brain barrier penetration	P-gp substrate	Blood-brain barrier penetration	P-gp substrate	BBB score
**4**	No	No	0.018	No	2.89
**5**	No	No	0.029	No	2.87
**6**	No	No	0.069	Yes	2.47
**7**	No	No	0.038	No	2.78
**8**	No	No	0.075	No	2.76
**9**	No	No	0.089	Yes	2.36
**10**	No	No	0.102	No	2.78
**11**	No	No	0.112	No	2.76
**12**	No	No	0.112	No	2.36
**15**	Yes	No	0.049	No	3.55

2.3. Gastrointestinal absorption calculations using PreADMET tool.

Table S3 lists the results derived from the calculations regarding the gastrointestinal absorption of the studied compounds, using the PreADMET tool.

Table S3 - Gastrointestinal absorption calculations using the PreADMET tool.

**Compound**	**GI absorption**	**% protein binding**
**4**	High	82.49

https://doi.org/10.3390/ijms27104443

**5**	High	75.08
**6**	High	80.56
**7**	High	65.92
**8**	High	59.36
**9**	High	66.78
**10**	High	59.61
**11**	High	53.55
**12**	High	62.49
**15**	High	71.76
**Warfarin**	High	90.40

2.4.Metabolism calculations using GLORYx platform.

The following Table S4 depicts the structures of the proposed metabolites of each compound (in decreasing order or probability – written in brackets) as produced by the GLORYx platform.

Table S4 - Metabolism prediction using the GLORYx platform.

Compound	Metabolites		
	1st	2nd	3rd
**4**	**(0.40)**	**(0.40)**	**(0.37)**
**5**	**(0.52)**	**(0.52)**	**(0.48)**
**6**	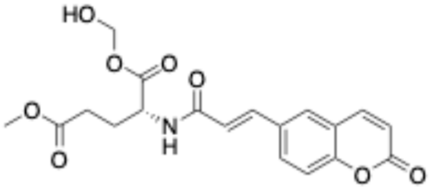 **(0.53)**	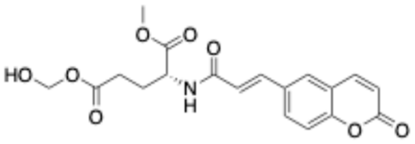 **(0.53)**	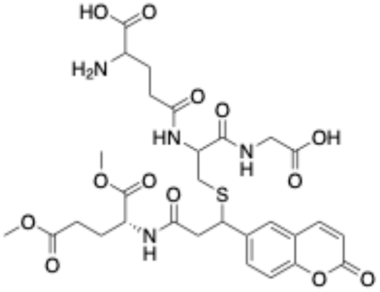 **(0.38)**

https://doi.org/10.3390/ijms27104443

**7**	**(0.41)**	**(0.40)**	**(0.36)**
**8**	**(0.51)**	**(0.51)**	**(0.36)**
**9**	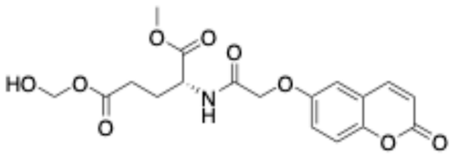 **(0.53)**	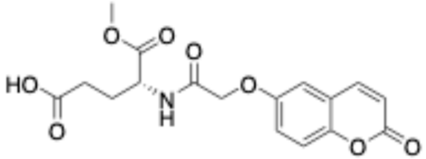 **(0.53)**	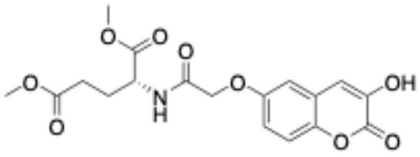 **(0.36)**
**10**	**(0.40)**	**(0.40)**	**(0.36)**
**11**	**(0.51)**	**(0.51)**	**(0.38)**
**12**	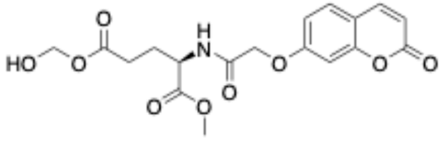	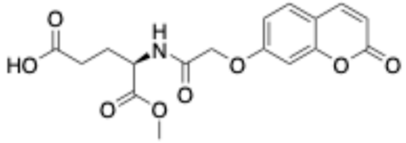	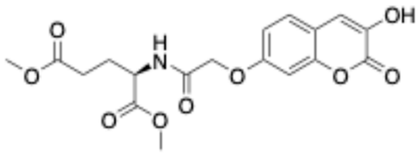

	**(0.53)**	**(0.53)**	**(0.38)**
**15**	**(0.40)**	**(0.40)**	**(0.36)**
**Ibuprofen**	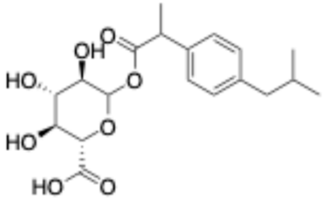 **(0.99)**	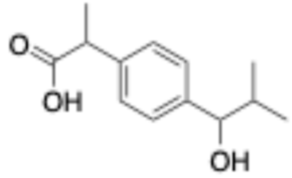 **(0.61)**	**-**
**Zileuton**	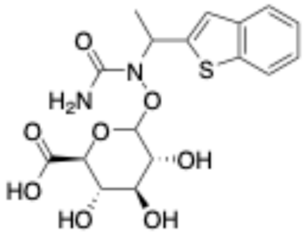 **(0.81)**	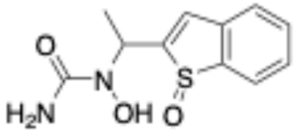 **(0.54)**	**-**

2.5.Cytochrome P-450 inhibitory calculations using the CypRules tool.

Table S5 shows the data retrieved from the CypRule tool regarding the cytochrome P-450 inhibitory calculations.

https://doi.org/10.3390/ijms27104443

Table S5 - Cytochrome P-450 inhibitory calculations using the tool CypRules.

Compound	CYP1A2	CYP2C19	CYP2C9	CYP2D6	CYP3A4
**4**	No inhibition	No inhibition	No inhibition	No inhibition	No inhibition
**5**	No inhibition	No inhibition	No inhibition	No inhibition	No inhibition
**6**	No inhibition	No inhibition	No inhibition	No inhibition	No inhibition
**7**	No inhibition	No inhibition	No inhibition	No inhibition	No inhibition
**8**	No inhibition	No inhibition	No inhibition	No inhibition	No inhibition
**9**	No inhibition	No inhibition	No inhibition	No inhibition	No inhibition
**10**	No inhibition	No inhibition	No inhibition	No inhibition	No inhibition
**11**	No inhibition	No inhibition	No inhibition	No inhibition	No inhibition
**12**	No inhibition	No inhibition	No inhibition	No inhibition	No inhibition
**15**	No inhibition	No inhibition	No inhibition	No inhibition	No inhibition

2.6. Hepatotoxicity evaluation using the LiverTox Workspace platform.

https://doi.org/10.3390/ijms27104443

In Tables S6 and S7, the hepatotoxicity data regarding the interaction of the studied molecules with several transporters are shown, as retrieved by the LiverTox Workspace platform. [**BSEP inhib. =** Bile Salt Export Pump inhibition (or ATP-binding cassette, sub-family B member 11 inhibition), **BSEP trans. =** Bile Salt Export Pump transport (or ATP-binding cassette, sub-family B member 11 transport), **P-gp inhib**. = Permeability glycoprotein inhibition, **P-gp trans.** = Permeability glycoprotein transport, **MRP4 inhib. =** Multidrug Resistance-associated protein 4 inhibition**, MRP4 trans. =** Multidrug Resistance-associated protein 2 transport, **MRP3 inhib. =** Multidrug Resistance-associated protein 3 inhibition, **MRP3 trans.** = Multidrug Resistance-associated protein 3 transport, **BCRP inhib.** = Breast Cancer Resistance Protein inhibition, **BCRP trans. =** Breast Cancer Resistance Protein transport, **OATP1B1 inhib**.= Organic Anion Transporting Polypeptide 1 inhibition, **OATP1B3 inhib**. = Organic Anion Transporting Polypeptide 3 inhibition].

Table S6 - Hepatotoxicity data using the LiverTox platform.

Compound	BSEP inhib.	BSEP trans.	P-gp inhib.	P-gp trans.	MRP4 inhib.	MRP2 trans.	MRP3 inhib.	MRP3 trans.
**4**	Negative	Positive	Negative	Negative	Positive	Positive	Negative	Negative
**5**	Negative	Positive	Negative	Negative	Positive	Positive	Negative	Negative
**6**	Negative	Positive	Negative	Negative	Positive	Positive	Negative	Negative
**7**	Negative	Negative	Negative	Negative	Positive	Positive	Negative	Negative
**8**	Negative	Negative	Positive	Negative	Positive	Positive	Negative	Negative
**9**	Negative	Negative	Negative	Negative	Positive	Positive	Negative	Negative
**10**	Negative	Negative	Negative	Negative	Positive	Positive	Negative	Negative
**11**	Negative	Negative	Negative	Negative	Positive	Positive	Negative	Negative
**12**	Negative	Negative	Negative	Negative	Positive	Positive	Negative	Negative
**15**	Negative	Positive	Negative	Negative	Negative	Positive	Negative	Negative
Ximelagatran	Positive	Negative	Positive	Positive	Positive	Positive	Positive	Negative

Table S7 - Hepatotoxicity data retrieved from the LiverTox Workspace platoform.

Compound	BCRP inhib.	BCRP trans.	OATP1B1 inhib.	OATP1B3 inhib.	Drug-induced liver damage	Hyperbilirubinemia	Cholestasis
**4**	Negative	Positive	Negative	Negative	Positive	Negative	Negative
**5**	Negative	Positive	Negative	Negative	Positive	Negative	Positive

**6**	Negative	Positive	Negative	Negative	Positive	Negative	Positive
**7**	Negative	Positive	Negative	Negative	Positive	Positive	Negative
**8**	Negative	Positive	Negative	Negative	Positive	Positive	Positive
**9**	Negative	Positive	Negative	Negative	Positive	Positive	Positive
**10**	Negative	Positive	Negative	Negative	Positive	Positive	Negative
**11**	Negative	Positive	Negative	Negative	Positive	Positive	Positive
**12**	Negative	Positive	Negative	Negative	Positive	Positive	Positive
**15**	Negative	Positive	Negative	Negative	Positive	Negative	Positive
Ximelagatran	Positive	Positive	Positive	Positive	Positive	Negative	Positive

Table S8. Predicted Properties of compound 4

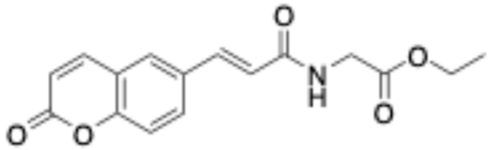

Resume of the properties from different platforms

Platform	Properties
Molinspiration	milogP = 2.06, TPSA = 85.61, MW = 301.30, Hbond acceptor = 6, Hbond donor = 1, violations = 0, Rotatable bonds = 6, MV = 265.52
PreADMET	BBB penetration = 0.018, Pgp substrate = NO, Absorption = High, % protein binding = 82.49%
Molsoft	BBB score = 2.89
CypRules	Does not inhibit any CYP isoforms
SwissADME	BBB penetration = NO, Pgp substrate = NO<br>*I ran a new study for this compound on 22/3:*

https://doi.org/10.3390/ijms27104443

	Fsp3 = 0.19, Solubility (ESOL) = 0.501 mg/mL (soluble), molecular refractivity = 80.90, Bioavailability = 0.55, leadlikeness = Yes (by Lipinski, Veber, Ghose, Egan, Muegge rules)
QED score	0.836
pKa	*pKa = 12.6 in pH = 9 or pH = 8.5 the molecule is uncharged* 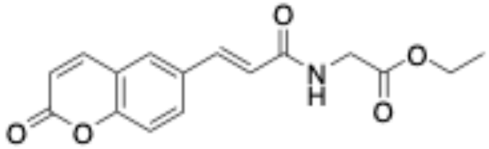

References

The reference list can be found included within the main text.

Disclaimer/Publisher's Note: The statements, opinions and data contained in all publications are solely those of the individual author(s) and contributor(s) and not of MDPI and/or the editor(s). MDPI and/or the editor(s) disclaim responsibility for any injury to people or property resulting from any ideas, methods, instructions or products referred to in the content.
